# Crystal structure and Hirshfeld surface analysis of 3-eth­oxy-1-ethyl-6-nitro­quinoxalin-2(1*H*)-one

**DOI:** 10.1107/S2056989023007624

**Published:** 2023-09-08

**Authors:** Seqqat Yousra, Lhoussaine El Ghayati, Tuncer Hökelek, Fouad Ouazzani Chahdi, Joel T. Mague, Youssef Kandri Rodi, Nada Kheira Sebbar

**Affiliations:** a Laboratory of Applied Organic Chemistry, Faculty of Science and Technology, Sidi Mohammed Ben Abdullah University, Route d’Immouzzer, BP 2202, Fez, Morocco; b Laboratory of Heterocyclic Organic Chemistry, Medicines Science Research Center, Pharmacochemistry Competence Center, Mohammed V University in Rabat, Faculty of Sciences, Morocco; c Department of Physics, Hacettepe University, 06800 Beytepe, Ankara, Türkiye; d Department of Chemistry, Tulane University, New Orleans, LA 70118, USA; e Laboratory of Organic and physical Chemistry, Applied Bioorganic Chemistry Team, Faculty of Sciences, Ibn Zohr University, Agadir, Morocco; University of Aberdeen, United Kingdom

**Keywords:** crystal structure, hydrogen bond, π-stacking, quinoxaline

## Abstract

The asymmetric unit of the title compound, C_12_H_13_N_3_O_4_, consists of two mol­ecules differing to a small degree in their conformations. In the crystal, layers of mol­ecules are connected by weak C—H⋯O hydrogen bonds and slipped π-stacking inter­actions. These layers lie parallel to (10  ) and are stacked along the normal to that plane. Hirshfeld surface analysis indicates that the most important contributions for the crystal packing arise from H⋯H (43.5%) and H⋯O/O⋯H (30.8%) contacts. The density functional theory (DFT) optimized structure of the title compound at the B3LYP/ 6–311 G(d,p) level agrees well with the experimentally determined mol­ecular structure in the solid state.

## Abstract 2

The asymmetric unit of the title compound consists of two mol­ecules differing to a small degree in their conformations. In the crystal, layers of mol­ecules are connected by weak C—H⋯O hydrogen bonds and slipped π-stacking inter­actions.

## 1. Chemical context

Quinoxaline derivatives made up of a fused benzene ring and pyrazine ring constitute an important class of heterocyclic compounds which, even when part of a complex mol­ecule, possess a wide spectrum of biological activities (Abad *et al.*, 2020 ▸). Quinoxaline derivatives have been synthesized by several methods (Chen *et al.*, 2021 ▸; Ramli *et al.*, 2010 ▸) and possess inter­esting properties such as anti-bacterial (Ammar *et al.*, 2020 ▸), anti-inflammatory (Meka & Chintakunta, 2023 ▸), anti­cancer (Jain *et al.*, 2019 ▸) and kinase inhibition (Oyallon *et al.*, 2018 ▸). They have also been studied as fungicides, herbicides and insecticides (Pathakumari *et al.*, 2020 ▸). 
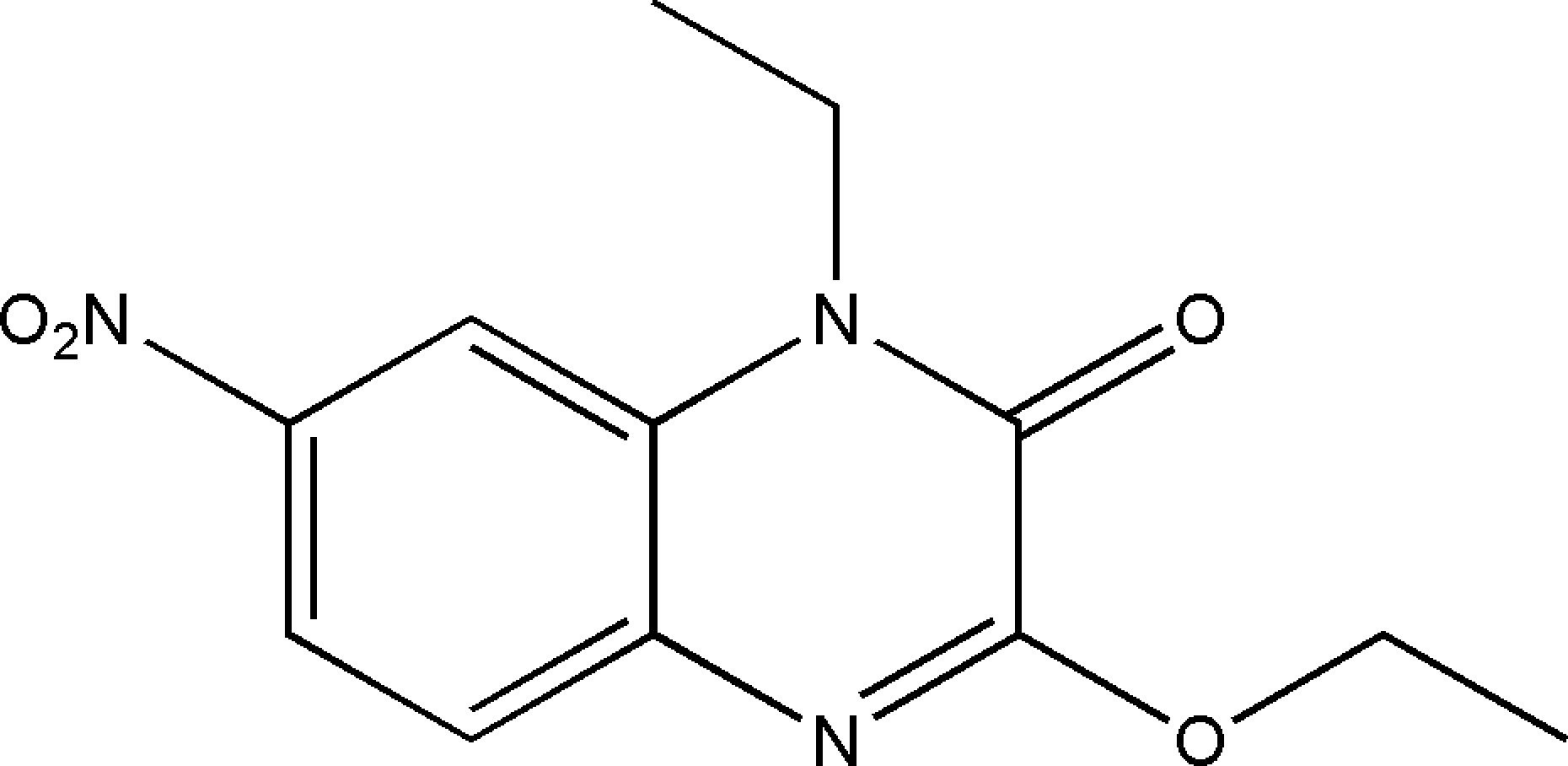


In a continuation of our ongoing research in this area (Abad *et al.*, 2020 ▸), we have synthesized the title compound (I) by reacting ethyl bromide with 6-nitro-1,4-di­hydro­quinoxaline-2,3-dione and potassium carbonate in the presence of tetra-*n*-butyl ammonium bromide as catalyst. We report herein the synthesis, crystal structure and Hirshfeld surface analysis and the density functional theory (DFT) computational calculations carried out at the B3LYP/6–311G(d,p) level for comparing with the experimentally determined mol­ecular structure in the solid state of the title compound.

## 2. Structural commentary

**Figure 1:**
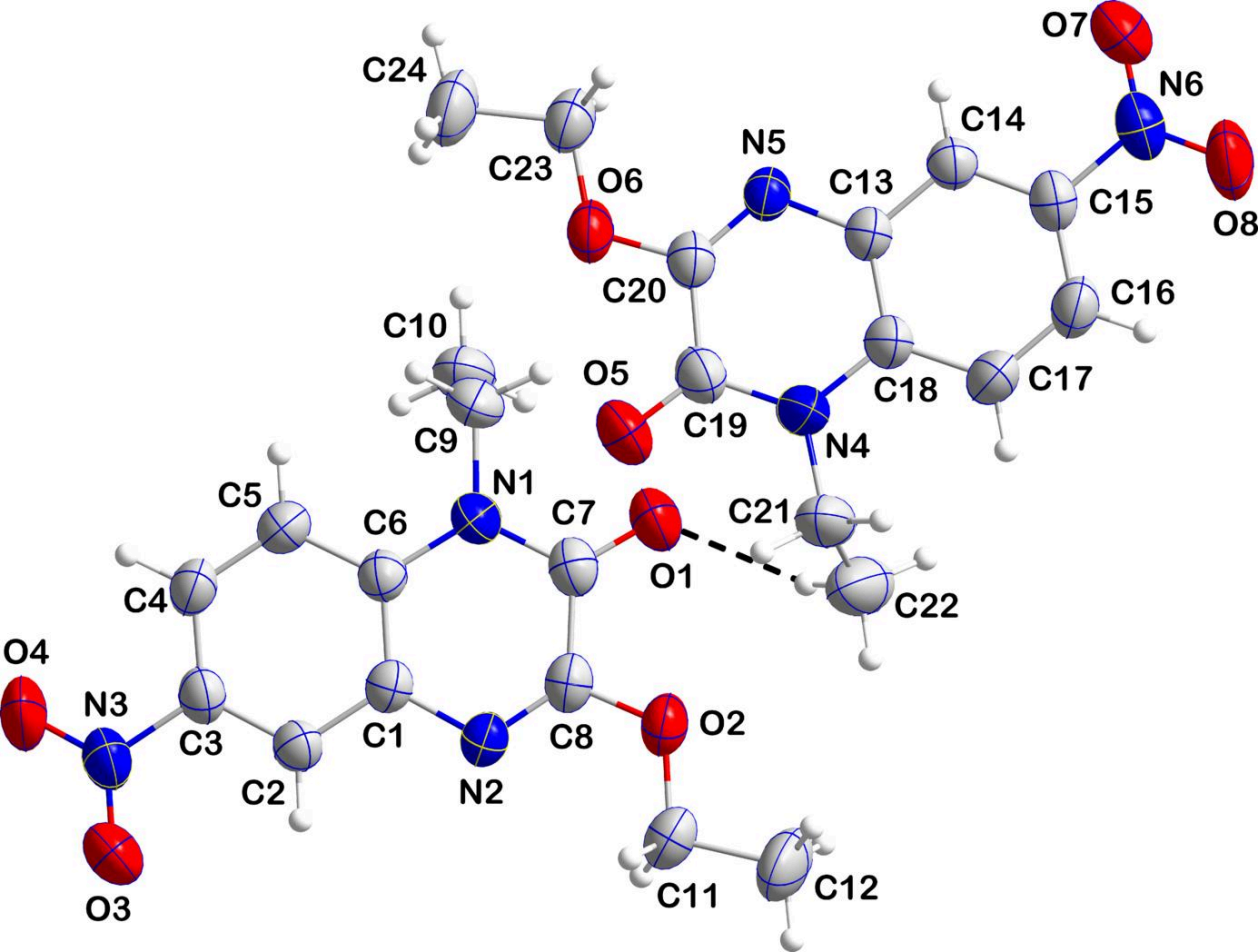
The asymmetric unit with labelling scheme and 50% probability ellipsoids. Only the major component of the disordered ethyl group is shown and the C—H⋯O hydrogen bond is depicted by a dashed line.

The asymmetric unit of (I) consists of two independent mol­ecules containing C1 and C13 differing to small degrees in conformation (Fig. 1 ▸). The most notable difference is a disorder of the C21/C22 ethyl group attached to N4 in one mol­ecule while in the other mol­ecule, there is no disorder. In one mol­ecule, the dihedral angle between the mean planes of the C1–C6 and the C1/C6/N1/C7/C8/N2 rings is 4.69 (4)° while in the second mol­ecule the corresponding angle between the C13–C18 and C13/C18/N4/C19/C20/N5 rings is 3.17 (5)°. In addition, the heterocyclic ring in the C1 mol­ecule deviates more from planarity than does that in the C13 mol­ecule (r.m.s. deviation of the fitted atoms = 0.034 Å for the former and 0.017 Å for the latter).

## 3. Supra­molecular features

**Figure 2:**
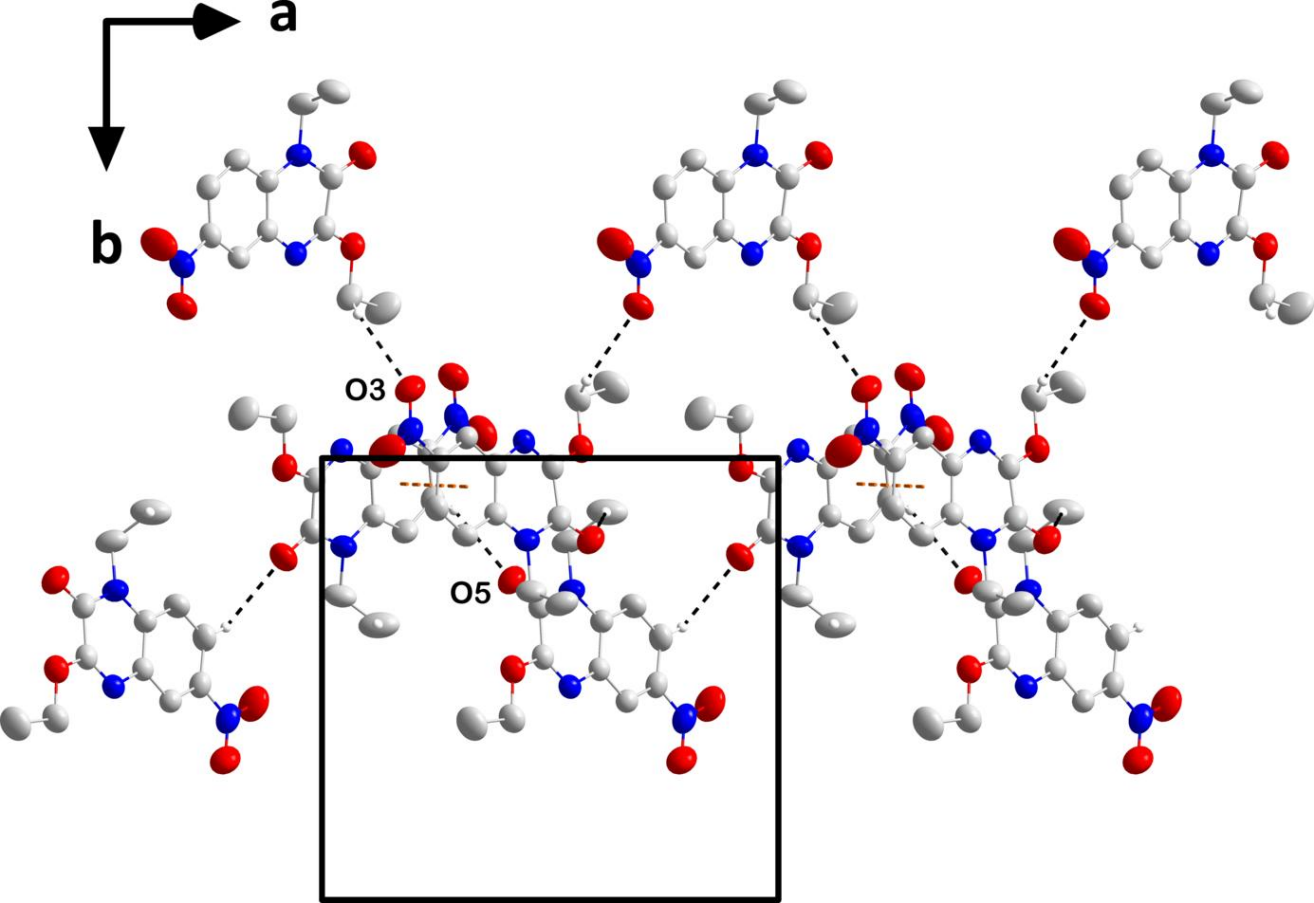
Portions of the chains formed by mol­ecules containing C1 (top) and C13 (bottom) viewed along the *c*-axis direction with C—H⋯O hydrogen bonds and slipped π-stacking inter­actions depicted, respectively, by black and orange dashed lines. Non-inter­acting hydrogen atoms are omitted for clarity.

**Figure 3:**
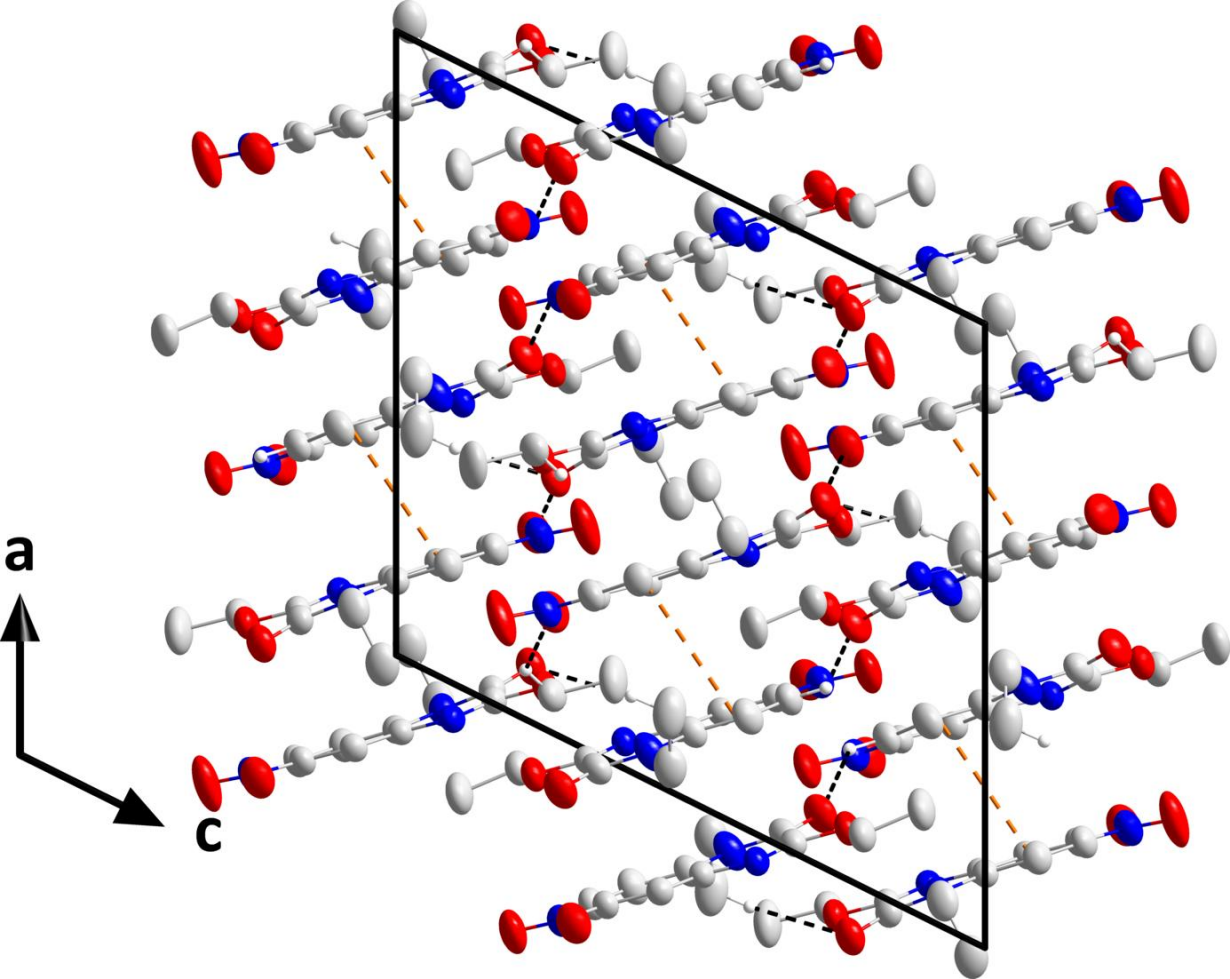
Packing viewed along the *b*-axis direction with C—H⋯O hydrogen bonds and slipped π-stacking inter­actions depicted, respectively, by black and orange dashed lines. Non-inter­acting hydrogen atoms are omitted for clarity.

In the crystal, C11—H11*A*⋯O1 hydrogen bonds (Table 1 ▸) form chains of the C1 mol­ecules extending parallel to (10 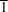 ) while C16—H16⋯O5 hydrogen bonds (Table 1 ▸) form parallel chains from the C13 mol­ecule (Fig. 2 ▸). The chains are cross-linked by C22—H22*A*⋯O1 hydrogen bonds (Table 1 ▸) and slipped π-stacking inter­actions between C1–C6 rings related by the symmetry operation *x* + 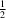 , −*y* + 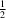 , *z* + 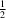 [centroid–centroid separation = 3.7558 (9) Å, dihedral angle = 8.65 (8)°, slippage = 1.15 Å] into layers lying parallel to (10 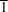 ) (Fig. 2 ▸). The layers stack along the normal to (10 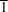 ) with unexceptional van der Waals contacts (Fig. 3 ▸).

## 4. Hirshfeld surface analysis and DFT calculations

**Figure 4:**
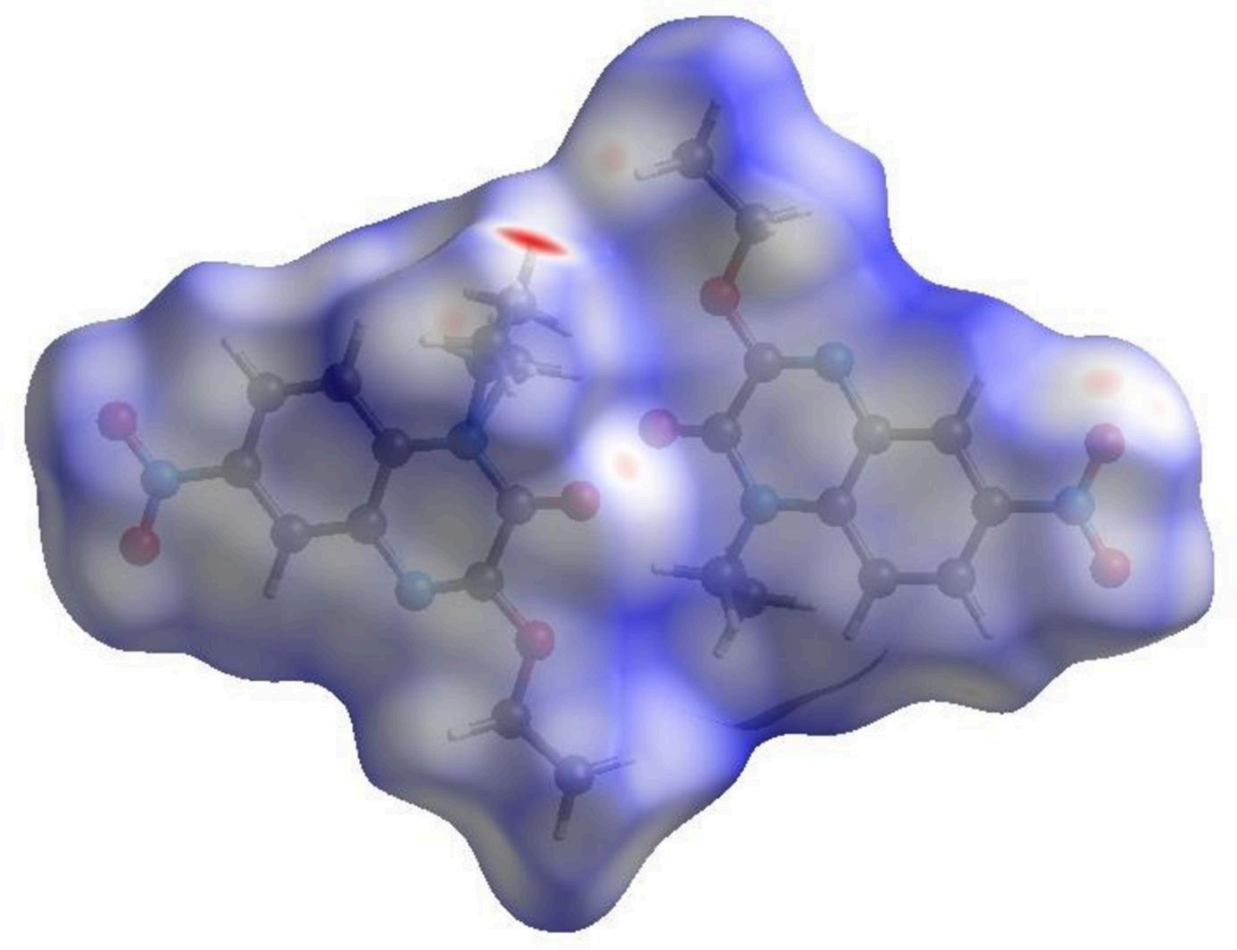
View of the three-dimensional Hirshfeld surface of the title compound, plotted over *d*_norm_ in the range −0.45 to 1.40 a.u.

**Figure 5:**
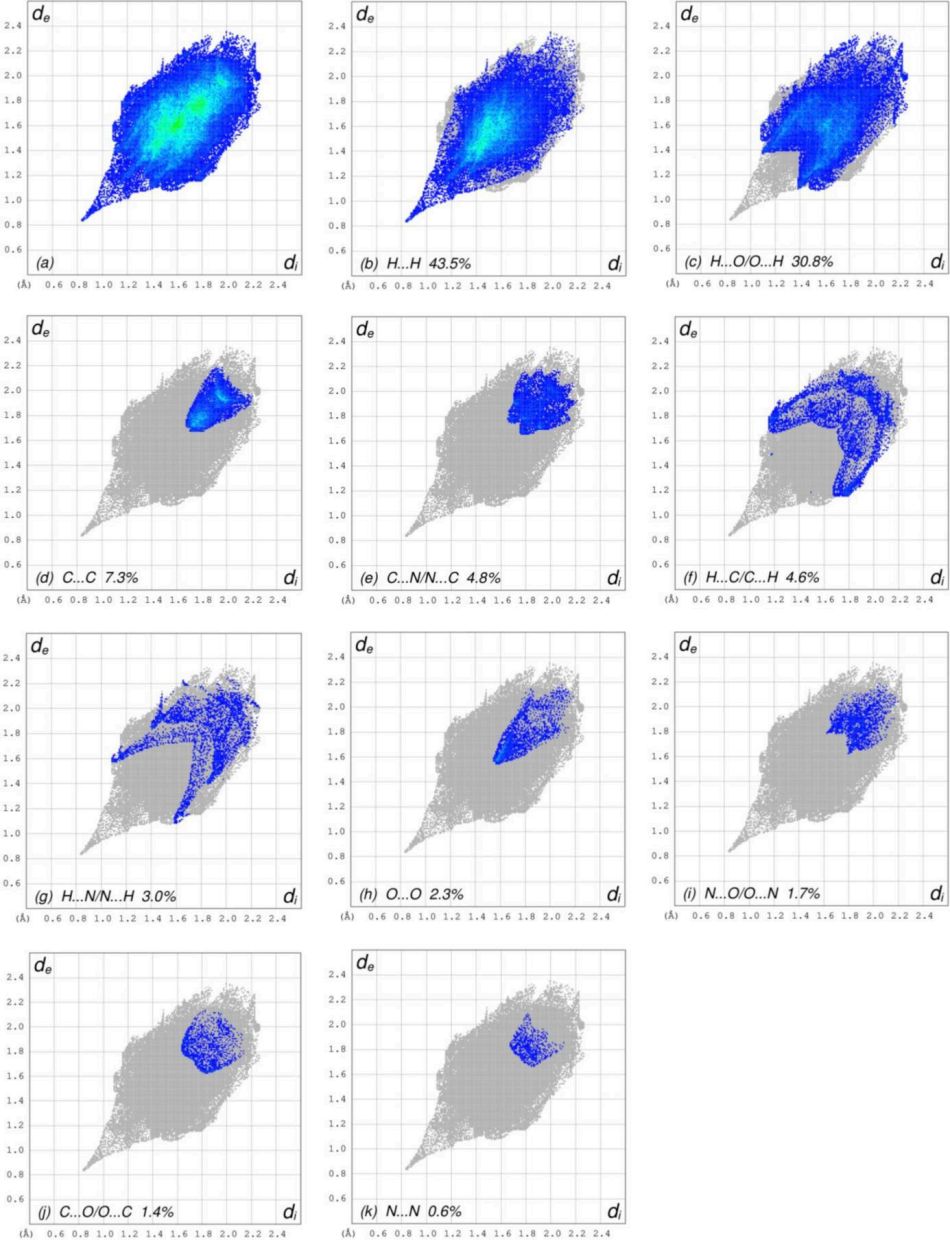
The full two-dimensional fingerprint plots for the title compound, showing (*a*) all inter­actions, and delineated into (*b*) H⋯H, (*c*) H⋯O/O⋯H, (*d*) C⋯C,(*e*) C⋯N/N⋯C, (*f*) H⋯C/C⋯H, (*g*) H⋯N/N⋯H, (*h*) O⋯O, (i) N⋯O/O⋯N, (*j*) C⋯O/O⋯C and (*k*) N⋯N inter­actions. The *d*_i_ and *d*_e_ values are the closest inter­nal and external distances (in Å) from given points on the Hirshfeld surface.

To further visualize the inter­molecular inter­actions in the crystal of (I), a Hirshfeld surface (HS) analysis was carried out using *Crystal Explorer 17.5* (Turner *et al.*, 2017 ▸). The Hirshfeld surface plotted over *d*_norm_ is shown in Fig. 4 ▸. The overall two-dimensional fingerprint plot, Fig. 5 ▸ *a*, and those delineated into H⋯H, H⋯O/O⋯H, C⋯C, C⋯N/N⋯C, H⋯C/C⋯H, H⋯N/N⋯H, O⋯O, N⋯O/O⋯N, C⋯O/O⋯C and N⋯N contacts (McKinnon *et al.*, 2007 ▸) are illustrated in Fig. 5 ▸ *b*–*k*, respectively, together with their relative contributions to the Hirshfeld surface. The most important contact type is H⋯H, contributing 43.5% to the overall crystal packing, which is reflected in Fig. 5 ▸ *b* as widely scattered points of high density due to the large hydrogen content of the mol­ecule, with the tip at *d*_e_ = *d*_i_ = 0.83 Å. The pair of spikes in the fingerprint plot delineated into H⋯O/O⋯H contacts (Fig. 5 ▸ *c*; 30.8% contribution to the HS) has an almost symmetric distribution of points with the tips at *d*_e_ + *d*_i_ = 2.46 Å. The C⋯C contacts (Fig. 5 ▸ *d*), appearing as a bullet-shaped distribution of points, make a contribution of 7.3% to the HS with the tip at *d*_e_ = *d*_i_ = 1.65 Å. The tiny wing pair of C⋯N/N⋯C contacts (Fig. 5 ▸ *e*) with a 4.8% contribution to the HS are viewed at *d*_e_ + *d*_i_ = 3.42 Å. In the absence of C—H⋯π inter­actions, the pair of characteristic wings in the fingerprint plot delineated into H⋯C/C⋯H contacts with the tips at *d*_e_ + *d*_i_ = 2.83 Å, Fig. 5 ▸ *f*, make a 4.6% contribution to the HS. The spikes of H⋯N/N⋯H contacts (Fig. 5 ▸ *g*) with 3.0% contribution to the HS are viewed at *d*_e_ + *d*_i_ = 2.66 Å. The O⋯O contacts (Fig. 5 ▸ *h*) with an arrow-shaped distribution of points with the tip at *d*_e_ = *d*_i_ = 1.57 Å make a contribution of 2.3% to the HS. The tiny spikes of N⋯O/O⋯N contacts (Fig. 5 ▸ *i*) with 1.7% contribution to the HS are viewed at *d*_e_ + *d*_i_ = 3.43 Å. Finally, the C⋯O/O⋯C (Fig. 5 ▸ *j*) and N⋯N (Fig. 5 ▸ *k*) contacts contribute 1.4% and 0.6%, respectively, to the HS.

The optimized structure of (I) in the gas phase was generated *via* a density functional theory (DFT) calculation using the standard B3LYP functional and 6-311 G(d,p) basis-set calculations (Becke, 1993 ▸) as implemented in *GAUSSIAN 09*. Table S1 shows that the theoretically calculated geometric parameters are in good agreement with the corresponding ones obtained from the X–ray analysis. The frontier orbitals (HOMO and LUMO) of (I) are depicted in Fig. S1. The electron density of the HOMO is mostly distributed in the quinoline moiety, while that of the LUMO is mostly distributed over the phenyl ring. The HOMO–LUMO energy gap is 4.39 eV.

## 5. Database survey

A survey of the Cambridge Structural Database (CSD) (Version 5.42, last update February 2023; Groom *et al.*, 2016 ▸) using search fragment **II** yielded 25 hits. 
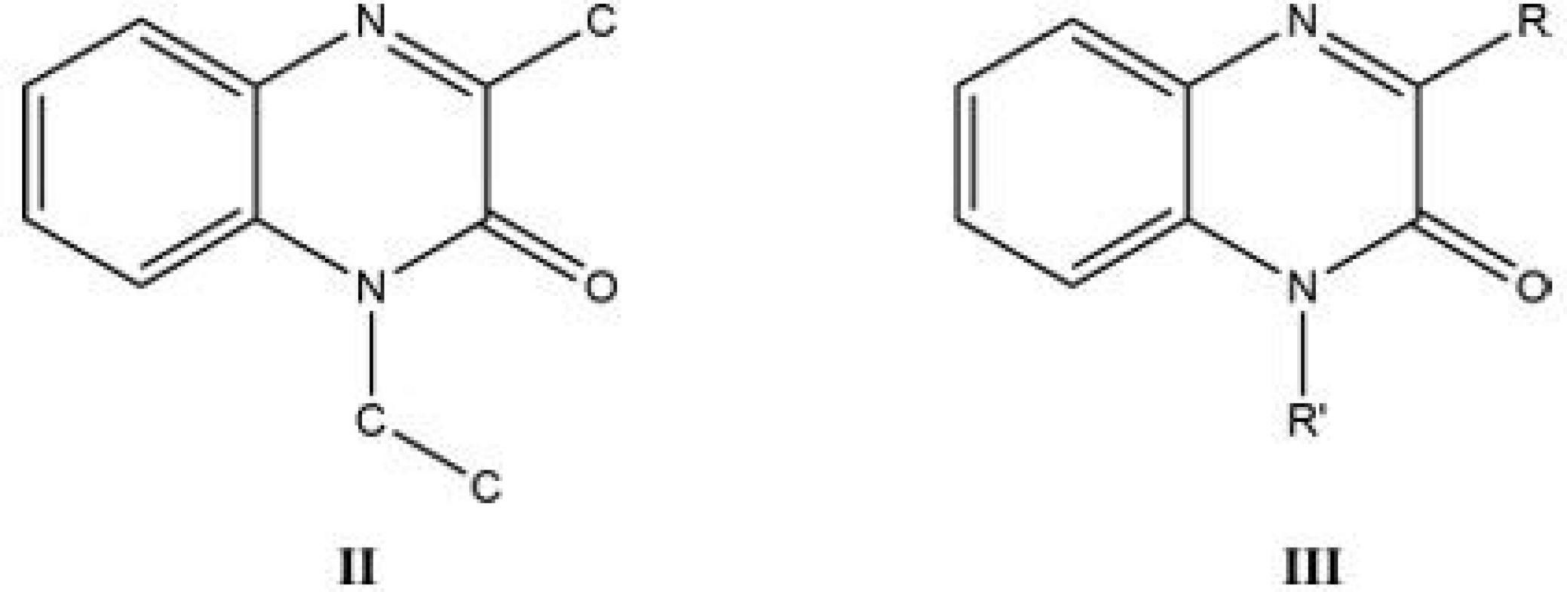


Of these hits, those most similar to the title mol­ecule have the formula **III** with *R* = Me and *R*′ = CH_2_CO_2_H (CSD refcode DEZJAW; Missioui *et al.*, 2018 ▸), benzyl (DUSHUV; Ramli *et al.*, 2010 ▸), with *R* = CF_3_ and *R*′ = *i*-Bu (DUBPUO; Wei *et al.*, 2019 ▸) and with *R* = Ph and *R*′ = CH_2_(cyclo-CHCH_2_O) benzyl (PUGGII; Benzeid *et al.*, 2009 ▸). In the majority of hits, the di­hydro­quinoxaline ring is essentially planar with the dihedral angle between the constituent rings being less than 1° or having the nitro­gen atom bearing the exocyclic substituent less than 0.03 Å from the mean plane of the remaining nine atoms.

## 6. Synthesis and crystallization

To a solution of 6-nitro-1,4-di­hydro­quinoxaline-2,3-dione (2 mmol), potassium carbonate (4 mmol) and tetra-*n*-butyl­ammonium­bromide (0.2 mmol) in di­methyl­formamide (DMF) (20 ml) were added ethyl bromide (4 mmol), and the mixture was then left to stir for 12 h at room temperature. Following salt filtration, the solution was evaporated at a low pressure, and the resulting residue was dissolved in di­chloro­methane. The organic phase was then dried over Na_2_SO_4_ and concentrated. The resulting mixture was chromatographed using a silica gel column with hexa­ne/ethyl­acetate as the eluent (4/1). Single crystals of the title compound suitable for X-ray analysis were obtained by slow evaporation of a methanol solution.

## 7. Refinement

Crystal data, data collection and structure refinement details are summarized in Table 2 ▸. H atoms were positioned geometrically (C—H = 0.94–0.98 Å) and refined as riding with *U*_iso_(H) = 1.2–1.5*U*_eq_(C).

## Supplementary Material

Crystal structure: contains datablock(s) global, I. DOI: 10.1107/S2056989023007624/hb8072sup1.cif

Structure factors: contains datablock(s) I. DOI: 10.1107/S2056989023007624/hb8072Isup2.hkl

Supporting information file. DOI: 10.1107/S2056989023007624/hb8072Isup3.cdx

Supporting information file. DOI: 10.1107/S2056989023007624/hb8072Isup4.cml

CCDC reference: 2292321

Additional supporting information:  crystallographic information; 3D view; checkCIF report

## Figures and Tables

**Table 1:** Hydrogen-bond geometry (Å, °)

*D*—H⋯*A*	*D*—H	H⋯*A*	*D*⋯*A*	*D*—H⋯*A*
C11—H11*A*⋯O3^i^	0.98	2.59	3.250 (2)	125
C16—H16⋯O5^ii^	0.94	2.58	3.2624 (19)	130
C22—H22*A*⋯O1	0.97	2.55	3.487 (4)	162

Symmetry codes: (i) 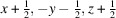 ; (ii) 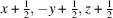 .

**Table 2:** Experimental details

Crystal data	
Chemical formula	C_12_H_13_N_3_O_4_
*M*_r_	263.25
Crystal system, space group	Monoclinic, *P*2_1_/*n*
Temperature (K)	240
*a*, *b*, *c* (Å)	14.4848 (3), 12.5663 (2), 15.2708 (3)
β (°)	116.424 (1)
*V* (Å^3^)	2489.20 (8)
*Z*	8
Radiation type	Cu *K*α
μ (mm^−1^)	0.91
Crystal size (mm)	0.25 × 0.16 × 0.09

Data collection	
Diffractometer	Bruker D8 VENTURE PHOTON 3 CPAD
Absorption correction	Multi-scan (*SADABS*; Krause *et al.*, 2015 ▸)
*T*_min_, *T*_max_	0.81, 0.93
No. of measured, independent and observed [*I* > 2σ(*I*)] reflections	66682, 5082, 4257
*R*_int_	0.031
(sin θ/λ)_max_ (Å^−1^)	0.625

Refinement	
*R*[*F* ^2^ > 2σ(*F* ^2^)], *wR*(*F* ^2^), *S*	0.047, 0.137, 1.04
No. of reflections	5082
No. of parameters	355
No. of restraints	2
H-atom treatment	H-atom parameters constrained
Δρ_max_, Δρ_min_ (e Å^−3^)	0.31, −0.24

Computer programs: *APEX4* and *SAINT* (Bruker, 2021 ▸), *SHELXT* (Sheldrick, 2015*a* ▸), *SHELXL2018* (Sheldrick, 2015*b* ▸), *DIAMOND* (Brandenburg & Putz, 2012 ▸) and *SHELXTL* (Sheldrick, 2008 ▸).

## supplementary crystallographic information

### Crystal data

**Table:**

C_12_H_13_N_3_O_4_	*F*(000) = 1104
*M**_r_* = 263.25	*D*_x_ = 1.405 Mg m^−^^3^
Monoclinic, *P*2_1_/*n*	Cu *K*α radiation, λ = 1.54178 Å
*a* = 14.4848 (3) Å	Cell parameters from 9131 reflections
*b* = 12.5663 (2) Å	θ = 3.6–74.5°
*c* = 15.2708 (3) Å	µ = 0.91 mm^−^^1^
β = 116.424 (1)°	*T* = 240 K
*V* = 2489.20 (8) Å^3^	Plate, colourless
*Z* = 8	0.25 × 0.16 × 0.09 mm

### Data collection

**Table:**

Bruker D8 VENTURE PHOTON 3 CPAD diffractometer	5082 independent reflections
Radiation source: INCOATEC IµS micro–focus source	4257 reflections with *I* > 2σ(*I*)
Mirror monochromator	*R*_int_ = 0.031
Detector resolution: 7.3910 pixels mm^-1^	θ_max_ = 74.6°, θ_min_ = 3.5°
φ and ω scans	*h* = −18→18
Absorption correction: multi-scan (*SADABS*; Krause *et al.*, 2015)	*k* = −15→15
*T*_min_ = 0.81, *T*_max_ = 0.93	*l* = −19→19
66682 measured reflections	

### Refinement

**Table:**

Refinement on *F*^2^	Primary atom site location: dual
Least-squares matrix: full	Secondary atom site location: difference Fourier map
*R*[*F*^2^ > 2σ(*F*^2^)] = 0.047	Hydrogen site location: inferred from neighbouring sites
*wR*(*F*^2^) = 0.137	H-atom parameters constrained
*S* = 1.04	*w* = 1/[σ^2^(*F*_o_^2^) + (0.0675*P*)^2^ + 0.623*P*] where *P* = (*F*_o_^2^ + 2*F*_c_^2^)/3
5082 reflections	(Δ/σ)_max_ < 0.001
355 parameters	Δρ_max_ = 0.31 e Å^−^^3^
2 restraints	Δρ_min_ = −0.24 e Å^−^^3^

### Special details

**Table:**

Experimental. The diffraction data were obtained from 18 sets of frames, each of width 0.5° in ω or φ, collected with scan parameters determined by the "strategy" routine in *APEX4*. The scan time was θ-dependent and ranged from 5 to 15 sec/frame.
Geometry. All esds (except the esd in the dihedral angle between two l.s. planes) are estimated using the full covariance matrix. The cell esds are taken into account individually in the estimation of esds in distances, angles and torsion angles; correlations between esds in cell parameters are only used when they are defined by crystal symmetry. An approximate (isotropic) treatment of cell esds is used for estimating esds involving l.s. planes.
Refinement. Refinement of F^2^ against ALL reflections. The weighted R-factor wR and goodness of fit S are based on F^2^, conventional R-factors R are based on F, with F set to zero for negative F^2^. The threshold expression of F^2^ > 2sigma(F^2^) is used only for calculating R-factors(gt) etc. and is not relevant to the choice of reflections for refinement. R-factors based on F^2^ are statistically about twice as large as those based on F, and R- factors based on ALL data will be even larger. H-atoms attached to carbon were placed in calculated positions (C—H = 0.95 - 0.99 Å). All were included as riding contributions with isotropic displacement parameters 1.2 - 1.5 times those of the attached atoms. The C21-C22 ethyl group is disordered over two resolved sites in a 0.584 (3)/0.416 (3) ratio and the two components were refined with restraints that their geometries be comparable

### Fractional atomic coordinates and isotropic or equivalent isotropic displacement parameters (Å^2^)

**Table:**

	*x*	*y*	*z*	*U*_iso_*/*U*_eq_	Occ. (<1)
O1	0.58896 (9)	0.18517 (10)	0.72886 (8)	0.0684 (3)	
O2	0.56752 (8)	−0.02104 (9)	0.75322 (7)	0.0577 (3)	
O3	0.19338 (10)	−0.15645 (11)	0.26502 (10)	0.0782 (4)	
O4	0.14323 (14)	−0.01613 (14)	0.17867 (10)	0.1109 (6)	
N1	0.44938 (10)	0.18526 (10)	0.57903 (9)	0.0511 (3)	
N2	0.44267 (8)	−0.03747 (10)	0.59520 (8)	0.0467 (3)	
N3	0.19358 (11)	−0.06088 (12)	0.25626 (10)	0.0642 (4)	
C1	0.38082 (10)	0.01493 (11)	0.50836 (9)	0.0426 (3)	
C2	0.31774 (10)	−0.04604 (11)	0.42806 (10)	0.0464 (3)	
H2	0.317239	−0.120587	0.432885	0.056*	
C3	0.25591 (10)	0.00398 (12)	0.34124 (10)	0.0492 (3)	
C4	0.25135 (11)	0.11315 (13)	0.33181 (11)	0.0541 (3)	
H4	0.206429	0.145274	0.272425	0.065*	
C5	0.31383 (12)	0.17440 (12)	0.41099 (11)	0.0539 (3)	
H5	0.311584	0.248970	0.405576	0.065*	
C6	0.38049 (10)	0.12651 (11)	0.49926 (10)	0.0450 (3)	
C7	0.52019 (11)	0.13682 (13)	0.66320 (10)	0.0517 (3)	
C8	0.50543 (10)	0.02007 (12)	0.66644 (10)	0.0473 (3)	
C9	0.45860 (15)	0.30158 (13)	0.57188 (13)	0.0666 (4)	
H9A	0.488141	0.332644	0.637598	0.080*	
H9B	0.389883	0.332289	0.534364	0.080*	
C10	0.52561 (17)	0.32914 (14)	0.52314 (14)	0.0766 (5)	
H10A	0.497112	0.297461	0.458485	0.115*	
H10B	0.594651	0.301914	0.561957	0.115*	
H10C	0.528319	0.405843	0.517508	0.115*	
C11	0.56054 (13)	−0.13524 (13)	0.76522 (11)	0.0605 (4)	
H11A	0.579346	−0.174311	0.719964	0.073*	
H11B	0.490108	−0.155018	0.752061	0.073*	
C12	0.63413 (19)	−0.16070 (19)	0.86897 (13)	0.0921 (7)	
H12A	0.632603	−0.236554	0.880035	0.138*	
H12B	0.614122	−0.122158	0.912869	0.138*	
H12C	0.703298	−0.139877	0.881165	0.138*	
O5	0.41594 (10)	0.27601 (10)	0.77918 (9)	0.0750 (4)	
O6	0.41831 (8)	0.47798 (9)	0.73392 (7)	0.0588 (3)	
O7	0.78801 (11)	0.68361 (11)	1.19824 (10)	0.0799 (4)	
O8	0.85032 (11)	0.55613 (13)	1.30161 (9)	0.0905 (5)	
N4	0.54742 (11)	0.29975 (11)	0.93216 (11)	0.0677 (4)	
N5	0.54324 (8)	0.51731 (9)	0.88781 (8)	0.0468 (3)	
N6	0.79426 (10)	0.58951 (13)	1.21966 (10)	0.0627 (4)	
C13	0.60770 (10)	0.47806 (11)	0.98025 (9)	0.0438 (3)	
C14	0.66831 (10)	0.54983 (11)	1.05191 (10)	0.0461 (3)	
H14	0.665103	0.622919	1.037718	0.055*	
C15	0.73319 (10)	0.51310 (12)	1.14396 (10)	0.0498 (3)	
C16	0.74255 (12)	0.40606 (14)	1.16725 (11)	0.0578 (4)	
H16	0.789513	0.382691	1.229824	0.069*	
C17	0.68202 (12)	0.33456 (13)	1.09731 (12)	0.0621 (4)	
H17	0.686887	0.261628	1.112391	0.075*	
C18	0.61299 (11)	0.36931 (12)	1.00359 (11)	0.0518 (3)	
C19	0.47782 (11)	0.33450 (13)	0.84112 (11)	0.0564 (4)	
C20	0.48407 (10)	0.45019 (12)	0.82464 (10)	0.0483 (3)	
C21	0.5397 (4)	0.1885 (2)	0.9655 (3)	0.0725 (10)	0.584 (3)
H21A	0.545104	0.190628	1.031782	0.087*	0.584 (3)
H21B	0.472838	0.157386	0.921877	0.087*	0.584 (3)
C22	0.6251 (3)	0.1219 (3)	0.9644 (3)	0.0948 (10)	0.584 (3)
H22A	0.621554	0.123323	0.899417	0.142*	0.584 (3)
H22B	0.617965	0.049216	0.981755	0.142*	0.584 (3)
H22C	0.690994	0.150227	1.011130	0.142*	0.584 (3)
C21A	0.5585 (6)	0.1809 (2)	0.9319 (4)	0.0725 (10)	0.416 (3)
H21C	0.534530	0.152573	0.865601	0.087*	0.416 (3)
H21D	0.629601	0.157939	0.972557	0.087*	0.416 (3)
C22A	0.4881 (5)	0.1506 (4)	0.9761 (4)	0.0948 (10)	0.416 (3)
H22D	0.418325	0.172893	0.933020	0.142*	0.416 (3)
H22E	0.510762	0.185404	1.039032	0.142*	0.416 (3)
H22F	0.489736	0.074101	0.984864	0.142*	0.416 (3)
C23	0.41646 (13)	0.58928 (14)	0.70810 (11)	0.0630 (4)	
H23A	0.481697	0.609561	0.707899	0.076*	
H23B	0.405551	0.634521	0.754941	0.076*	
C24	0.32870 (16)	0.60100 (19)	0.60766 (13)	0.0839 (6)	
H24A	0.324461	0.674367	0.586453	0.126*	
H24B	0.264740	0.581156	0.609230	0.126*	
H24C	0.340219	0.555057	0.562429	0.126*	

### Atomic displacement parameters (Å^2^)

**Table:**

	*U*^11^	*U*^22^	*U*^33^	*U*^12^	*U*^13^	*U*^23^
O1	0.0666 (7)	0.0702 (7)	0.0516 (6)	−0.0133 (6)	0.0110 (5)	−0.0110 (5)
O2	0.0527 (6)	0.0681 (7)	0.0402 (5)	0.0037 (5)	0.0097 (4)	0.0060 (5)
O3	0.0808 (8)	0.0657 (8)	0.0680 (8)	−0.0162 (6)	0.0149 (6)	−0.0151 (6)
O4	0.1185 (12)	0.0978 (11)	0.0523 (7)	−0.0217 (9)	−0.0196 (7)	0.0053 (7)
N1	0.0575 (7)	0.0460 (6)	0.0439 (6)	−0.0005 (5)	0.0173 (5)	−0.0048 (5)
N2	0.0428 (6)	0.0522 (6)	0.0410 (6)	0.0024 (5)	0.0151 (5)	0.0053 (5)
N3	0.0571 (7)	0.0682 (9)	0.0499 (7)	−0.0110 (6)	0.0081 (6)	−0.0044 (6)
C1	0.0391 (6)	0.0471 (7)	0.0400 (6)	0.0016 (5)	0.0162 (5)	0.0023 (5)
C2	0.0431 (7)	0.0463 (7)	0.0456 (7)	−0.0025 (5)	0.0159 (6)	0.0014 (5)
C3	0.0422 (7)	0.0564 (8)	0.0417 (7)	−0.0047 (6)	0.0120 (6)	−0.0020 (6)
C4	0.0495 (7)	0.0581 (8)	0.0430 (7)	0.0032 (6)	0.0100 (6)	0.0086 (6)
C5	0.0583 (8)	0.0459 (7)	0.0497 (8)	0.0044 (6)	0.0170 (7)	0.0054 (6)
C6	0.0448 (7)	0.0469 (7)	0.0407 (7)	0.0022 (5)	0.0167 (5)	−0.0017 (5)
C7	0.0499 (7)	0.0587 (8)	0.0431 (7)	−0.0020 (6)	0.0177 (6)	−0.0057 (6)
C8	0.0420 (6)	0.0581 (8)	0.0389 (6)	0.0022 (6)	0.0154 (5)	0.0031 (6)
C9	0.0817 (11)	0.0442 (8)	0.0587 (9)	0.0015 (7)	0.0177 (8)	−0.0096 (7)
C10	0.0962 (14)	0.0537 (9)	0.0664 (11)	−0.0152 (9)	0.0242 (10)	0.0010 (8)
C11	0.0582 (9)	0.0647 (10)	0.0496 (8)	0.0100 (7)	0.0160 (7)	0.0133 (7)
C12	0.1129 (17)	0.0938 (15)	0.0472 (9)	0.0270 (13)	0.0154 (10)	0.0162 (9)
O5	0.0672 (7)	0.0703 (8)	0.0665 (7)	−0.0164 (6)	0.0108 (6)	−0.0165 (6)
O6	0.0545 (6)	0.0687 (7)	0.0395 (5)	0.0039 (5)	0.0084 (4)	−0.0010 (5)
O7	0.0814 (8)	0.0699 (8)	0.0713 (8)	−0.0128 (7)	0.0185 (7)	−0.0192 (6)
O8	0.0821 (9)	0.1119 (12)	0.0456 (6)	−0.0207 (8)	−0.0003 (6)	−0.0032 (7)
N4	0.0618 (8)	0.0444 (7)	0.0686 (9)	−0.0033 (6)	0.0035 (7)	0.0003 (6)
N5	0.0449 (6)	0.0510 (6)	0.0392 (6)	0.0030 (5)	0.0142 (5)	0.0021 (5)
N6	0.0524 (7)	0.0794 (10)	0.0474 (7)	−0.0110 (6)	0.0144 (6)	−0.0106 (6)
C13	0.0399 (6)	0.0477 (7)	0.0403 (7)	0.0019 (5)	0.0149 (5)	0.0016 (5)
C14	0.0445 (7)	0.0477 (7)	0.0438 (7)	−0.0011 (5)	0.0175 (6)	0.0002 (5)
C15	0.0420 (7)	0.0613 (9)	0.0408 (7)	−0.0039 (6)	0.0138 (6)	−0.0030 (6)
C16	0.0495 (8)	0.0668 (9)	0.0446 (7)	0.0032 (7)	0.0097 (6)	0.0087 (7)
C17	0.0602 (9)	0.0518 (8)	0.0576 (9)	0.0039 (7)	0.0111 (7)	0.0118 (7)
C18	0.0463 (7)	0.0472 (7)	0.0500 (8)	−0.0001 (6)	0.0107 (6)	0.0014 (6)
C19	0.0471 (7)	0.0573 (9)	0.0537 (8)	−0.0030 (6)	0.0125 (6)	−0.0081 (7)
C20	0.0422 (7)	0.0564 (8)	0.0413 (7)	0.0035 (6)	0.0139 (5)	−0.0013 (6)
C21	0.094 (2)	0.0477 (12)	0.063 (3)	−0.0111 (13)	0.0230 (18)	0.0014 (13)
C22	0.147 (3)	0.0596 (16)	0.0650 (16)	0.0060 (19)	0.0352 (18)	0.0036 (13)
C21A	0.094 (2)	0.0477 (12)	0.063 (3)	−0.0111 (13)	0.0230 (18)	0.0014 (13)
C22A	0.147 (3)	0.0596 (16)	0.0650 (16)	0.0060 (19)	0.0352 (18)	0.0036 (13)
C23	0.0613 (9)	0.0723 (10)	0.0449 (8)	0.0092 (8)	0.0142 (7)	0.0088 (7)
C24	0.0837 (13)	0.1014 (15)	0.0454 (9)	0.0179 (11)	0.0098 (9)	0.0116 (9)

### Geometric parameters (Å, º)

**Table:**

O1—C7	1.2156 (18)	O8—N6	1.2234 (19)
O2—C8	1.3308 (16)	N4—C19	1.376 (2)
O2—C11	1.456 (2)	N4—C18	1.3906 (19)
O3—N3	1.2084 (19)	N4—C21A	1.503 (3)
O4—N3	1.2178 (19)	N4—C21	1.509 (3)
N1—C7	1.3789 (18)	N5—C20	1.2809 (18)
N1—C6	1.3938 (17)	N5—C13	1.3909 (16)
N1—C9	1.4762 (19)	N6—C15	1.4597 (19)
N2—C8	1.2856 (18)	C13—C14	1.3880 (19)
N2—C1	1.3920 (16)	C13—C18	1.406 (2)
N3—C3	1.4563 (18)	C14—C15	1.3771 (19)
C1—C2	1.3886 (18)	C14—H14	0.9400
C1—C6	1.4088 (19)	C15—C16	1.382 (2)
C2—C3	1.3777 (19)	C16—C17	1.372 (2)
C2—H2	0.9400	C16—H16	0.9400
C3—C4	1.378 (2)	C17—C18	1.402 (2)
C4—C5	1.378 (2)	C17—H17	0.9400
C4—H4	0.9400	C19—C20	1.485 (2)
C5—C6	1.3975 (19)	C21—C22	1.500 (5)
C5—H5	0.9400	C21—H21A	0.9800
C7—C8	1.487 (2)	C21—H21B	0.9800
C9—C10	1.503 (3)	C22—H22A	0.9700
C9—H9A	0.9800	C22—H22B	0.9700
C9—H9B	0.9800	C22—H22C	0.9700
C10—H10A	0.9700	C21A—C22A	1.500 (5)
C10—H10B	0.9700	C21A—H21C	0.9800
C10—H10C	0.9700	C21A—H21D	0.9800
C11—C12	1.499 (2)	C22A—H22D	0.9700
C11—H11A	0.9800	C22A—H22E	0.9700
C11—H11B	0.9800	C22A—H22F	0.9700
C12—H12A	0.9700	C23—C24	1.502 (2)
C12—H12B	0.9700	C23—H23A	0.9800
C12—H12C	0.9700	C23—H23B	0.9800
O5—C19	1.2181 (18)	C24—H24A	0.9700
O6—C20	1.3320 (16)	C24—H24B	0.9700
O6—C23	1.450 (2)	C24—H24C	0.9700
O7—N6	1.219 (2)		
			
C8—O2—C11	117.00 (12)	O7—N6—O8	122.89 (15)
C7—N1—C6	121.81 (12)	O7—N6—C15	118.61 (13)
C7—N1—C9	116.86 (12)	O8—N6—C15	118.49 (15)
C6—N1—C9	120.86 (12)	C14—C13—N5	118.24 (12)
C8—N2—C1	116.94 (12)	C14—C13—C18	119.19 (12)
O3—N3—O4	122.46 (15)	N5—C13—C18	122.57 (12)
O3—N3—C3	119.25 (13)	C15—C14—C13	119.46 (13)
O4—N3—C3	118.29 (15)	C15—C14—H14	120.3
C2—C1—N2	118.15 (12)	C13—C14—H14	120.3
C2—C1—C6	119.35 (12)	C14—C15—C16	122.21 (14)
N2—C1—C6	122.50 (12)	C14—C15—N6	119.06 (14)
C3—C2—C1	119.25 (13)	C16—C15—N6	118.73 (13)
C3—C2—H2	120.4	C17—C16—C15	118.77 (14)
C1—C2—H2	120.4	C17—C16—H16	120.6
C2—C3—C4	122.36 (13)	C15—C16—H16	120.6
C2—C3—N3	118.81 (13)	C16—C17—C18	120.60 (15)
C4—C3—N3	118.83 (13)	C16—C17—H17	119.7
C3—C4—C5	118.82 (13)	C18—C17—H17	119.7
C3—C4—H4	120.6	N4—C18—C17	122.21 (14)
C5—C4—H4	120.6	N4—C18—C13	118.09 (13)
C4—C5—C6	120.52 (14)	C17—C18—C13	119.69 (13)
C4—C5—H5	119.7	O5—C19—N4	123.26 (15)
C6—C5—H5	119.7	O5—C19—C20	122.57 (15)
N1—C6—C5	122.24 (13)	N4—C19—C20	114.17 (13)
N1—C6—C1	118.12 (12)	N5—C20—O6	122.66 (14)
C5—C6—C1	119.62 (12)	N5—C20—C19	126.01 (13)
O1—C7—N1	123.01 (15)	O6—C20—C19	111.33 (12)
O1—C7—C8	122.88 (14)	C22—C21—N4	109.1 (3)
N1—C7—C8	114.10 (12)	C22—C21—H21A	109.9
N2—C8—O2	122.29 (13)	N4—C21—H21A	109.9
N2—C8—C7	125.80 (12)	C22—C21—H21B	109.9
O2—C8—C7	111.89 (12)	N4—C21—H21B	109.9
N1—C9—C10	111.37 (14)	H21A—C21—H21B	108.3
N1—C9—H9A	109.4	C21—C22—H22A	109.5
C10—C9—H9A	109.4	C21—C22—H22B	109.5
N1—C9—H9B	109.4	H22A—C22—H22B	109.5
C10—C9—H9B	109.4	C21—C22—H22C	109.5
H9A—C9—H9B	108.0	H22A—C22—H22C	109.5
C9—C10—H10A	109.5	H22B—C22—H22C	109.5
C9—C10—H10B	109.5	C22A—C21A—N4	99.0 (4)
H10A—C10—H10B	109.5	C22A—C21A—H21C	112.0
C9—C10—H10C	109.5	N4—C21A—H21C	112.0
H10A—C10—H10C	109.5	C22A—C21A—H21D	112.0
H10B—C10—H10C	109.5	N4—C21A—H21D	112.0
O2—C11—C12	106.68 (15)	H21C—C21A—H21D	109.7
O2—C11—H11A	110.4	C21A—C22A—H22D	109.5
C12—C11—H11A	110.4	C21A—C22A—H22E	109.5
O2—C11—H11B	110.4	H22D—C22A—H22E	109.5
C12—C11—H11B	110.4	C21A—C22A—H22F	109.5
H11A—C11—H11B	108.6	H22D—C22A—H22F	109.5
C11—C12—H12A	109.5	H22E—C22A—H22F	109.5
C11—C12—H12B	109.5	O6—C23—C24	106.09 (15)
H12A—C12—H12B	109.5	O6—C23—H23A	110.5
C11—C12—H12C	109.5	C24—C23—H23A	110.5
H12A—C12—H12C	109.5	O6—C23—H23B	110.5
H12B—C12—H12C	109.5	C24—C23—H23B	110.5
C20—O6—C23	116.99 (12)	H23A—C23—H23B	108.7
C19—N4—C18	122.06 (13)	C23—C24—H24A	109.5
C19—N4—C21A	110.1 (3)	C23—C24—H24B	109.5
C18—N4—C21A	126.2 (3)	H24A—C24—H24B	109.5
C19—N4—C21	120.3 (2)	C23—C24—H24C	109.5
C18—N4—C21	116.4 (2)	H24A—C24—H24C	109.5
C20—N5—C13	116.92 (12)	H24B—C24—H24C	109.5
			
C8—N2—C1—C2	175.40 (12)	C18—C13—C14—C15	0.7 (2)
C8—N2—C1—C6	−4.29 (19)	C13—C14—C15—C16	1.9 (2)
N2—C1—C2—C3	−179.92 (12)	C13—C14—C15—N6	−178.12 (12)
C6—C1—C2—C3	−0.21 (19)	O7—N6—C15—C14	−2.0 (2)
C1—C2—C3—C4	−2.4 (2)	O8—N6—C15—C14	178.77 (14)
C1—C2—C3—N3	177.23 (12)	O7—N6—C15—C16	178.01 (15)
O3—N3—C3—C2	3.8 (2)	O8—N6—C15—C16	−1.2 (2)
O4—N3—C3—C2	−176.32 (17)	C14—C15—C16—C17	−2.7 (2)
O3—N3—C3—C4	−176.54 (15)	N6—C15—C16—C17	177.32 (14)
O4—N3—C3—C4	3.3 (2)	C15—C16—C17—C18	0.9 (3)
C2—C3—C4—C5	2.6 (2)	C19—N4—C18—C17	178.21 (16)
N3—C3—C4—C5	−177.08 (14)	C21A—N4—C18—C17	−17.8 (4)
C3—C4—C5—C6	−0.1 (2)	C21—N4—C18—C17	11.0 (3)
C7—N1—C6—C5	−175.02 (13)	C19—N4—C18—C13	−0.8 (2)
C9—N1—C6—C5	−3.1 (2)	C21A—N4—C18—C13	163.2 (3)
C7—N1—C6—C1	3.41 (19)	C21—N4—C18—C13	−168.0 (2)
C9—N1—C6—C1	175.31 (13)	C16—C17—C18—N4	−177.29 (16)
C4—C5—C6—N1	175.95 (13)	C16—C17—C18—C13	1.7 (2)
C4—C5—C6—C1	−2.5 (2)	C14—C13—C18—N4	176.54 (13)
C2—C1—C6—N1	−175.89 (12)	N5—C13—C18—N4	−3.0 (2)
N2—C1—C6—N1	3.80 (19)	C14—C13—C18—C17	−2.5 (2)
C2—C1—C6—C5	2.6 (2)	N5—C13—C18—C17	177.98 (13)
N2—C1—C6—C5	−177.72 (12)	C18—N4—C19—O5	−176.01 (16)
C6—N1—C7—O1	170.86 (14)	C21A—N4—C19—O5	17.7 (3)
C9—N1—C7—O1	−1.3 (2)	C21—N4—C19—O5	−9.3 (3)
C6—N1—C7—C8	−8.83 (19)	C18—N4—C19—C20	3.7 (2)
C9—N1—C7—C8	178.96 (13)	C21A—N4—C19—C20	−162.6 (3)
C1—N2—C8—O2	179.26 (11)	C21—N4—C19—C20	170.4 (2)
C1—N2—C8—C7	−2.2 (2)	C13—N5—C20—O6	179.47 (12)
C11—O2—C8—N2	−0.9 (2)	C13—N5—C20—C19	0.2 (2)
C11—O2—C8—C7	−179.69 (12)	C23—O6—C20—N5	0.5 (2)
O1—C7—C8—N2	−171.07 (14)	C23—O6—C20—C19	179.92 (13)
N1—C7—C8—N2	8.6 (2)	O5—C19—C20—N5	176.16 (15)
O1—C7—C8—O2	7.6 (2)	N4—C19—C20—N5	−3.6 (2)
N1—C7—C8—O2	−172.67 (12)	O5—C19—C20—O6	−3.2 (2)
C7—N1—C9—C10	91.55 (17)	N4—C19—C20—O6	177.06 (13)
C6—N1—C9—C10	−80.74 (18)	C19—N4—C21—C22	106.9 (3)
C8—O2—C11—C12	−177.81 (14)	C18—N4—C21—C22	−85.6 (3)
C20—N5—C13—C14	−176.30 (12)	C19—N4—C21A—C22A	−98.8 (4)
C20—N5—C13—C18	3.2 (2)	C18—N4—C21A—C22A	95.6 (5)
N5—C13—C14—C15	−179.71 (12)	C20—O6—C23—C24	−173.60 (14)

### Hydrogen-bond geometry (Å, º)

**Table:**

*D*—H···*A*	*D*—H	H···*A*	*D*···*A*	*D*—H···*A*
C11—H11*A*···O3^i^	0.98	2.59	3.250 (2)	125
C16—H16···O5^ii^	0.94	2.58	3.2624 (19)	130
C22—H22*A*···O1	0.97	2.55	3.487 (4)	162

Symmetry codes: (i) *x*+1/2, −*y*−1/2, *z*+1/2; (ii) *x*+1/2, −*y*+1/2, *z*+1/2.

### Comparison of the selected (X-ray and DFT) geometric data (Å, °)

**Table:**

Bonds/angles	X-ray	B3LYP/6-311G(d,p)
O1—C7	1.2156 (18)	1.2145
O2—C8	1.3308 (16)	1.3257
O2—C11	1.456 (2)	1.4490
O3—N3	1.2084 (19)	1.2236
O4—N3	1.2178 (19)	1.2254
C8—O2—C11	117.00 (12)	117.61
C7—N1—C6	121.81 (12)	122.08
C6—N1—C9	120.86 (12)	121.09
O3—N3—O4	122.46 (15)	123.09
O1—C7—N1	123.01 (15)	123.12

### Calculated energies.

**Table:**

Molecular Energy (a.u.) (eV)	Compound (I)
Total Energy *TE* (eV)	-25316,62
E_HOMO_ (eV)	-6.56
E_LUMO_ (eV)	-2.16
Gap *ΔE* (eV)	4.39
Dipole moment *µ* (Debye)	2.93
Ionisation potential *I* (eV)	6.56
Electron affinity *A*	2.16
Electronegativity *χ*	4.36
Hardness *η*	2.19
Softness *σ*	0.45
Electrophilicity index *ω*	-4.32
